# CFTR ion transport deficiency primes the epithelium for partial epithelial-mesenchymal transition in cystic fibrosis

**DOI:** 10.3389/fphar.2025.1655479

**Published:** 2025-08-20

**Authors:** Cláudia S. Rodrigues, Matilde Canto, Raquel Torres, Violeta Railean, Sofia S. Ramalho, Carlos M. Farinha, Ines Pankonien, Margarida D. Amaral

**Affiliations:** BioISI—Biosystems & Integrative Sciences Institute, Faculty of Sciences, University of Lisboa, Lisboa, Portugal

**Keywords:** cystic fibrosis, CFTR, P.Phe508del, p.Gly551Asp, epithelial-mesenchymal transition

## Abstract

**Introduction:**

Cystic fibrosis (CF) is a monogenic disease caused by mutations in the cystic fibrosis transmembrane conductance regulator (CFTR) gene, which encodes a Cl^−^/HCO_3_^−^ ion channel located at the apical plasma membrane (PM) of epithelial cells. CFTR dysfunction disrupts epithelial barrier integrity, drives progressive airway remodelling and has been associated with epithelial-to-mesenchymal transition (EMT), a process in which cells lose epithelial properties and acquire mesenchymal characteristics. We previously demonstrated that mutant CFTR directly drives partial EMT, independently of secondary events such as bacterial infection or inflammation.

**Methods:**

Here, we investigated whether PM localisation of CFTR alone is sufficient to preserve epithelial integrity or if its ion transport function is also required using polarized CF bronchial epithelial (CFBE) cells expressing wt-, p.Phe508del-, or p.Gly551Asp-CFTR. While p.Phe508del-CFTR is retained in the endoplasmic reticulum (ER) and fails to traffic to the PM, p.Gly551Asp-CFTR reaches the PM but lacks ion transport function. To this end we assessed transepithelial electrical resistance (TEER), cell proliferation, wound healing, and expression of epithelial and mesenchymal markers by Western blot and immunofluorescence.

**Results:**

The degree of mesenchymal phenotype was higher in cells expressing p.Phe508del-CFTR vs. those expressing PM localized but non-functional p.Gly551Asp-CFTR. This was evidenced by lower TEER, higher expression of mesenchymal markers (N-cadherin, vimentin), and lower E-/N-cadherin ratio. Furthermore, both CF cells displayed delayed wound healing compared to wt-CFTR cells, while only p.Phe508del-CFTR cells, but not p.Gly551Asp-CFTR cells, showed increased cell proliferation. Moreover, treatment with CFTR modulators (CFTRm) partially restored tight junction integrity by increasing claudin-1 levels and E-/N-cadherin ratio in both mutant cells. TGF-β1 treatment induced EMT in all three cell lines by decreasing epithelial markers (E-cadherin, cytokeratin 18, claudin-1) while increasing N-cadherin levels. However, mesenchymal marker vimentin increased only in CF cells, and more prominently in p.Phe508del-CFTR than in p.Gly551Asp-CFTR cells. Additionally, CFTR inhibition in wt-CFTR cells, partially mimicked p.Gly551Asp-CFTR behaviour, i.e., reduced claudin-1 levels.

**Discussion:**

Altogether, these findings demonstrate that the loss of CFTR ion transport, despite the physical presence of (nonfunctional) CFTR at the PM, is enough to trigger partial EMT. However, the severity of the EMT phenotype worsens when CFTR is absent from the PM while also increasing susceptibility to TGF-β1-triggered EMT. Moreover, CFTRm only partially reverse this CF EMT state, indicating that full epithelial integrity will likely require targeting additional factors.

## 1 Introduction

Cystic fibrosis (CF) is an autosomal recessive disease caused by mutations in the *cystic fibrosis transmembrane conductance regulator (CFTR)* gene. The CFTR protein primarily functions as an anion channel, facilitating the transport of chloride (Cl^−^) and bicarbonate (HCO_3_^−^) ions across the apical plasma membrane (PM) of epithelial cells (De Boeck and Amaral, 2016; Riordan et al., 1989). Consequently, CFTR dysfunction leads to epithelial surface dehydration, resulting in the accumulation of thick, sticky mucus that affects multiple organs, most notably the lungs, where respiratory disease remains the main cause of morbidity and mortality (Boucher, 2007).

Besides its primary role as an anion channel, CFTR has been implicated in various cellular processes, including epithelial differentiation/polarization, tissue regeneration, foetal development, proliferation and cancer (Amaral et al., 2020). It was demonstrated that functional CFTR is essential for the normal organization and function of tight junctions (TJs), thereby preserving epithelial tightness (LeSimple et al., 2010). Moreover, CFTR is required for the rapid regeneration of human airway surface epithelium after injury (Hajj et al., 2007; Trinh et al., 2012; Schiller et al., 2010). It also plays a critical role during foetal development, particularly in proximal airway morphogenesis (Meyerholz et al., 2010) and acts as a tumour suppressor by regulating epithelial cell proliferation (Than et al., 2016). Consistently, dysfunctional CFTR impairs the epithelial barrier by disrupting its structure and function, causes a delay in epithelial regeneration (Hajj et al., 2007), compromises wound healing (Hajj et al., 2007; Trinh et al., 2012; Schiller et al., 2010), and induces structural abnormalities during foetal development, such as defective tracheal cartilage and reduced respiratory function (Meyerholz et al., 2010). Furthermore, absence of functional CFTR has been linked to increased epithelial cell proliferation (Schiller et al., 2010), induction of epithelial-mesenchymal transition (EMT) (Zhang et al., 2013) and higher incidence of various cancer forms in people with CF (pwCF) and even CF carriers (Maisonneuve et al., 2013; Shi et al., 2021).

There is substantial evidence supporting that cancer predisposition in CF is linked to the role of functional CFTR in maintaining epithelial homeostasis and preventing EMT. EMT is a transcriptional reprogramming process of epithelial cells by which they acquire a mesenchymal phenotype and behaviour while losing epithelial features thus compromising epithelial integrity (Yang et al., 2020). Major EMT-associated transcription factors (TFs) include members of the SNAIL, ZEB and TWIST families (Dongre and Weinberg, 2019). During EMT, components of tight (TJ), adherens (AJ), gap (GJ) junctions, and desmosomes are inactivated, and the cytoskeleton acquires a less structured state. This results in apical-basal polarity loss, and changes in cell shape. In parallel, mesenchymal phenotype genes, e.g., those associated with the cytoskeleton (vimentin and αSMA), cell junctions (N-cadherin) and extracellular matrix proteins (ECM, collagen I and fibronectin), are activated (Dongre and Weinberg, 2019).

EMT is described to occur in three different types. Developmental EMT (Type I) is critical for tissue development and organogenesis. However, EMT also occurs in certain pathological conditions, e.g., inflammatory diseases (Type II) or cancer (Type III) (Vincent and Fuxe, 2017; Dongre and Weinberg, 2019). Cancer-related EMT contributes to tumour progression into invasive and metastatic disease (Vincent and Fuxe, 2017) while EMT type II is a driver of tissue remodelling and fibrosis in inflammatory diseases, as recently shown in several chronic lung diseases, including chronic obstructive pulmonary disease (COPD), idiopathic pulmonary fibrosis (IPF) and in CF (reviewed in (Rout-Pitt et al., 2018)). An important driver of EMT is TGF-β1 (transforming growth factor-beta) which is overexpressed in all these chronic airway diseases (Harris et al., 2011) and also widely demonstrated not only to be a modifier of CF lung disease severity (Drumm et al., 2005) but also described to degrade CFTR mRNA (Mitash et al., 2019), inhibit CFTR biogenesis and prevent functional rescue of p.Phe508del-CFTR (Snodgrass et al., 2013). In turn TGF-β1 signalling has been shown to be increased in CF (Harris et al., 2013; Nicola et al., 2019).

Along these lines, current evidence supports that EMT is rather a spectrum of dynamic yet stable states than a binary decision, with cells having a substantial degree of plasticity and expressing epithelial and mesenchymal phenotypes. In fact, most epithelial cells that have an EMT programme activated rarely advance to a full mesenchymal state, expressing simultaneously a mixture of markers (Dongre and Weinberg, 2019).

Emerging evidence suggests that CFTR is involved in preventing all three types of EMT [reviewed in (Amaral et al., 2020)]. Previously, our transcriptional profiling of CF airways revealed impaired epithelial differentiation and an EMT signature, even in the absence of inflammation (Clarke et al., 2015). We further demonstrated that EMT is active in CF native tissues, primary cells, and CFTR-mutant cell lines, characterized by disrupted epithelial structure, defective junctions, increased mesenchymal markers and EMT-TFs, hyperproliferation, and impaired repair (Quaresma et al., 2020). These changes are driven by TFs TWIST1 and YAP1 (Quaresma et al., 2022). Overall, CFTR appears to protect epithelial tissue from undergoing EMT by promoting epithelial differentiation and thus the integrity of the epithelium. However, the underlying molecular mechanisms remain unclear. Therefore, to further elucidate how CFTR dysfunction triggers EMT in CF we investigated the ability of two CFTR variants, each characterized by distinct molecular defects, to promote EMT: p.Phe508del (class II), which is retained in the endoplasmic reticulum and does not reach the PM, and p.Gly551Asp (class III), which reaches the PM but lacks ion channel function.

## 2 Materials and methods

### 2.1 Cell lines

CFBE41o- (Cystic Fibrosis Bronchial Epithelial) cells stably overexpressing wt- or p.Phe508del-CFTR were kindly provided by Dr. Zsuzsa Bebok, USA (Bebok et al., 2005). The cell line overexpressing p.Gly551Asp-CFTR was generated by lentiviral transduction of CFBE41o- cells. The p.Gly551Asp-CFTR cDNA was first cloned into the pLKO-puro plasmid, and the mutation was introduced by site-directed mutagenesis, as previously described (Canato et al., 2018). All cell lines were grown in Minimum Essential Medium Eagle (MEM) with Earl salts and L-glutamine (Corning, 10–010-CVR) supplemented with 10% (v/v) Foetal Bovine Serum (FBS) (Gibco, 10270), 1% Pen/Strep and puromycin (Sigma-Aldrich, P8833) at 5 μg/mL for selection. All cell lines were confirmed to be mycoplasma-free. To achieve polarization, cells were seeded on collagen IV-precoated Transwell permeable supports (12 mm insert, Corning 3470) at a density of 3 × 10^5^ cells per insert. The following day, the culture medium was changed from 10% to 2% (v/v) FBS to promote polarization. Polarization was assessed by monitoring transepithelial electrical resistance (TEER) over time using a volt-ohmmeter (Millicell-ERS, Millipore, MER5000001).

### 2.2 TGF-β1, CFTRinh-172 and CFTR modulator treatments

TGF-β1 treatment: Polarized CFBE cells were incubated 5 days after seeding with 15 ng/mL human TGF-β1 (PeproTech, 100–21) for 96 h, with treatment refreshed after 48h. A negative control with the solvent (10mM citric acid, pH 3 (Sigma-Aldrich, 251275) and 0.1% bovine serum albumin (BSA, Sigma-Aldrich, A9647)) was included in all experiments. CFTRinh-172 treatment: CFBE cells were incubated 3 days after seeding with 30 μM CFTRinh-172 (MedChemExpress, HY-16671) for 48 h, with treatment refreshed after 24 h. A negative control with the vehicle (DMSO) was included in all experiments. CFTRm treatment: For the treatments with CFTR modulators (3 μM VX-445 [SelleckChem, S8851], 5 μM VX-661 [SelleckChem, S7059], and/or 3 μM VX-770 [SelleckChem, S1144]), the compounds were added together with the FBS reduction (1 day after seeding). The treatment was refreshed 2 days later, and protein was collected 2 days after. All compounds were dissolved in DMSO, which was used as a vehicle control.

### 2.3 Proliferation assay

CFBE cells were seeded in 24-well plates pre-coated with collagen IV at a density of 50,000 cells per well. Cells were maintained in MEM supplemented with 10% (v/v) FBS, 1% Pen/Strep, and puromycin (5 μg/mL). Six hours after seeding, cells were harvested and counted (day 0), and the medium was renewed in the remaining wells. Cells were counted and medium changed every 2 days up to day 8. For each time point, 3 wells were analysed. The fold increase in cell number was calculated relative to the cell count on day 0.

### 2.4 Western blot (WB) analysis

Western blot (WB) analysis of cell lysates was performed as previously described (Quaresma et al., 2020). Cells were lysed with a buffer containing 31.25 mM Tris HCl (Sigma, 30721) pH 6.8; 1.5% (v/v) sodium dodecyl sulphate (SDS) (Gibco, 15553); 10% (v/v) glycerol (Sigma, 92025); 50mM dithiothreitol (DTT) (Sigma, D0632) and protease inhibitor cocktail (Roche, 11697498001). Benzonase (Sigma-Aldrich, E1014) 25 U/mL was also added to shear the DNA. SDS-PAGE was performed using polyacrylamide gels consisting of a 4% stacking and a 10% resolving gel. Proteins were transferred onto polyvinylidene difluoride (PVDF) membranes (Merck Millipore, IPVH00010) using a wet-transfer system. Membranes were blocked for 1h in 5% (w/v) non-fat milk (NFM) prepared in Tris buffer saline (TBS) supplemented with 0.1% Tween 20 (Fisher BioReagents, BP337–100). Primary antibodies were incubated overnight at 4 °C with gentle shaking. Horseradish peroxidase (HRP)-conjugated secondary antibodies were incubated for 1h at room temperature (RT). All antibodies were diluted in the blocking solution. Protein bands were visualized using the Chemidoc XRS+ system (Bio-Rad, 170–8265), and band intensities were quantified using Image Lab software (Bio-Rad, 170–9690), which calculates the integrated peak area. Signal intensities were normalized to loading controls (α-tubulin or GAPDH). A complete list of primary and secondary antibodies is available in (Quaresma et al., 2020).

### 2.5 Immunofluorescence assay

Polarized CFBE cells were fixed for 18 min with 4% (v/v) paraformaldehyde (PFA, Merck Millipore, 104003), followed by permeabilization and blocking for 30 min with 0.5% (v/v) triton X-100 (Amersham Biosciences, 17–1315–01) in 1% (w/v) bovine serum albumin (BSA) solution. Incubation with primary antibodies (ZO-1 (Invitrogen, 33-9100), E-cadherin (BD Biosciences, 610181), β-catenin (Abcam, ab32572) and vimentin (Abcam, ab92547)) was performed overnight at 4 °C. Antibody dilutions were done in 1% BSA solution. Secondary antibodies mix (Alexa Fluor 488 mouse IgG and alexa fluor 568 rabbit IgG (Life Technologies, A21202 and A10042, respectively) and nuclear dye (Hoechst) were incubated for 1h at RT. Filter sections were mounted in a mix of N-propylgallate (Sigma-Aldrich, P3130) and glycerol for microscopy (Merck, 104095). Imaging was performed with a Leica TCS SP8 confocal microscope coupled to a Hamamatsu Flash4 sCMOS camera, using HC Plan Apo 63×/1.4 objective. Software used for acquisition was Leica’s LAS X, and image processing was performed on ImageJ FIJI. FIJI was used to generate maximum image projections. Total fluorescence intensity was quantified using FIJI (ImageJ). A Z-projection (sum of slices) was applied to each image stack, and background subtraction was performed. A triangle thresholding method was used, as it provides greater sensitivity in the presence of high background signal. Nuclei were counted using FIJI by applying a consistent threshold and analysing particles. Fluorescence intensity was normalized to the number of nuclei to account for variations in cell density.

### 2.6 Wound healing assay

Fully polarized CFBE cells were mechanically injured by scraping a sterile P10 pipette tip across the cell monolayer. For cell wounding PBS was added to the apical side of the filters. After wounding the apical surface was washed twice with PBS to remove cell debris. Fresh media was added to the basolateral and apical side. Wound closure was monitored by live cell imaging (24 h, 37 °C, 5% CO2) with an automated Leica DMI6000B widefield microscope coupled to a Hamamatsu Flash4 sCMOS camera, using a HC PL/APO 10X/0.4 objective. Software used for acquisition was Leica’s LAS X, and image processing was performed on ImageJ FIJI. FIJI was used to segment, and measure wound area. Wound closure was then calculated as Wound size $\left(\%\right)=\left(\frac{At}{A0}\right)x100$ , where A_t_ is the area for a given time point and A0 is the initial wound area. Wound size was plotted as a function of time (h) and was used to calculate the rate (slope) of wound closure (%/h).

### 2.7 Statistical analyses

Statistical analyses were performed on GraphPad Prism 9.0 using Unpaired Student’s t-test or ANOVA followed by a Tukey’s test (post-hoc test), with p-value ≤ 0.05 considered as significantly different.

## 3 Results

### 3.1 Cells expressing p.Phe508del-CFTR are more mesenchymal than p.Gly551Asp-CFTR expressing cells

In a previous study, we demonstrated for the first time that mutant CFTR directly drives EMT, independently of secondary effects such as chronic inflammation or bacterial infection. These findings were based on p.Phe508del-CFTR, which disrupts both CFTR PM trafficking and channel gating (Class II) (Quaresma et al., 2020). However, it remained unclear whether correct PM localisation of CFTR would be sufficient to maintain epithelial integrity, or whether its ion transport function is also required. We, therefore, aimed to further dissect the contribution of specific CFTR defects on EMT activation in CF. To address this, we used CF human bronchial epithelial (CFBE) cell lines expressing either wild-type (wt), p.Phe508del-, or p.Gly551Asp-CFTR. While p.Phe508del-CFTR is retained in the ER, not reaching the PM (Class II), p.Gly551Asp-CFTR traffics to the PM but lacks channel function for Cl^−^/HCO_3_^−^ transport (Class III). These isogenic cell lines offer the advantage that any observed differences can be directly attributed solely to the specific CFTR variant expressed. Thus, this controlled setup allows us to study the differential impact of CFTR mislocalisation (p.Phe508del), independently of the effect associated with absence of ion transport (p.Gly551Asp).

**FIGURE 1:**
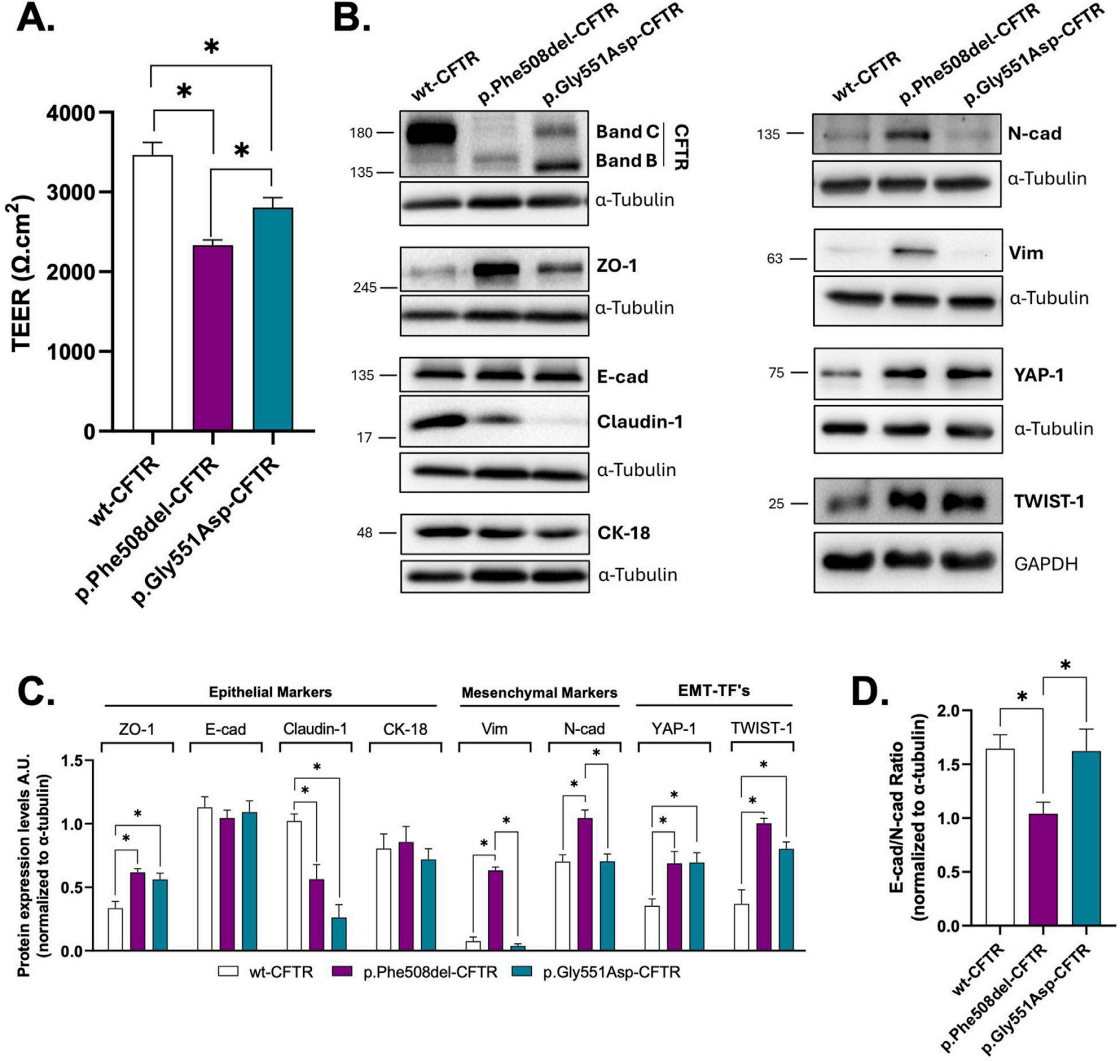
Characterization of the epithelial/mesenchymal status of wt-/p.Phe508del-/p.Gly551Asp-CFTR CFBE cells. **(A)** Transepithelial electrical resistance (TEER) measurements of polarized wt-, p.Phe508del- or p.Gly551Asp-CFTR CFBE cells. Data are presented as mean ± SEM. Asterisk (*) indicates significant difference (ANOVA, Tukey’s test, p < 0.05, n = 11). **(B)** Representative WB images showing the expression of epithelial (CFTR, Zonula Occludens-1 (ZO-1), E-cadherin (E-cad), Cytokeratin 18 (CK-18) and claudin-1) and mesenchymal (N-cadherin (N-cad), vimentin (Vim)) protein markers and EMT-TFs (TWIST-1 and YAP-1) in polarized CFBE cells. α-tubulin or GAPDH were used as loading control. **(C)** Quantification by densitometry of the protein expression detected by WB in B. Data is normalized to loading control, showed as arbitrary units (A.U.), mean ± SEM. Asterisk (*) indicates significant difference (ANOVA, Tukey’s test, p < 0.05, n = 8). **(D)** E-cadherin and N-cadherin ratio. Higher ratios indicate stronger epithelial phenotype, while lower ratios suggest a more mesenchymal phenotype. Data is normalized to loading control, showed as arbitrary units (A.U.), mean ± SEM. Asterisk (*) indicates significant difference (ANOVA, Tukey’s test, p < 0.05, n = 8).

We first assessed the epithelial barrier integrity by measuring transepithelial electrical resistance (TEER) on polarized CFBE cell monolayers stably expressing wt-, p.Phe508del-, or p.Gly551Asp-CFTR. TEER values were found to be significantly reduced in both CF cell lines compared to wt-CFTR cells, indicating that loss of CFTR ion transport (p.Gly551Asp-CFTR) already compromises epithelial barrier function (Figure 1A). Notably, p.Phe508del-CFTR cells exhibited lower TEER relative to p.Gly551Asp-CFTR cells, suggesting a stronger impact on barrier function with absence of CFTR at the PM. To further characterize the epithelial phenotype of the two cell lines, we next assessed the protein expression levels of epithelial and mesenchymal markers by Western blot (WB). As expected, both the mature (band C) and immature (band B) forms of CFTR were detected in p.Gly551Asp-CFTR as in wt-CFTR - expressing cells, whereas only the immature form was observed in p.Phe508del-CFTR cells, consistent with its ER retention (Figure 1B). Of note, functional analysis of CFTR in these cell lines performed by Ussing chamber is presented as Supplementary Data (Supplementary Figure S1). The levels of epithelial markers E-cadherin (E-cad) and cytokeratin 18 (CK18) were found to be comparable across all three cell lines (Figures 1B,C). However, differential expression levels were observed for epithelial tight junction proteins ZO-1 and claudin-1. While ZO-1 was upregulated, claudin-1 levels were decreased in both mutant CFTR cells compared to wt-CFTR cells (Figures 1B,C). Interestingly, the expression of mesenchymal markers N-cadherin (N-cad) and vimentin were found to be significantly elevated in p.Phe508del-CFTR cells, but not in p.Gly551Asp-CFTR cells, indicating a more pronounced EMT phenotype associated with the absence of CFTR at the PM (Figures 1B,C). Accordingly, the E-cadherin/N-cadherin ratio was significantly reduced in p.Phe508del- (but not in p.Gly551Asp-CFTR cells) relative to both wt-CFTR cells, further supporting a shift towards a stronger mesenchymal phenotype associated with absence of PM CFTR (Figure 1D). Next, we assessed the expression of EMT-associated transcription factors (EMTa-TFs), TWIST1 and YAP1 as we have previously shown that both are upregulated in CF cells (Quaresma et al., 2020; 2022). WB analysis revealed increased expression of these 2 EMTa-TFs in both CF cells in comparable levels, in comparison to wt-CFTR cells (Figures 1B,C). This indicates that loss of CFTR ion transport alone, due to impaired gating, can trigger the upregulation of EMT-promoting TFs.

**FIGURE 2:**
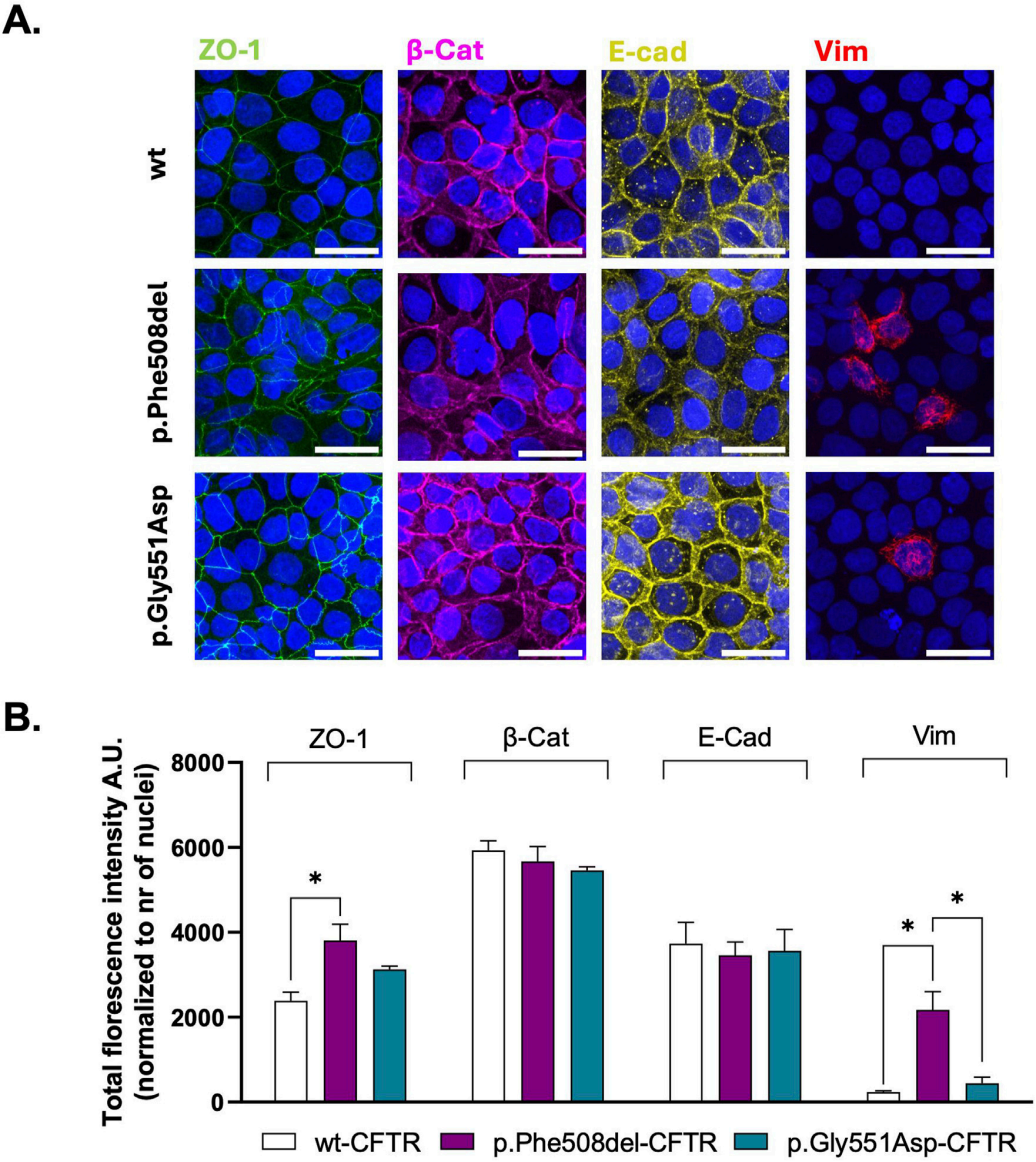
Assessment of the subcellular localization of epithelial and mesenchymal markers in polarized wt-, p.Phe508del-, and p.Gly551Asp-CFTR CFBE cells. **(A)** Representative immunofluorescence images showing the localization of epithelial (ZO-1, β-catenin [β-Cat], E-cadherin [E-cad]) and mesenchymal (vimentin [Vim]) markers in polarized wt-, p.Phe508del-, or p.Gly551Asp-CFTR CFBE cells. Nuclei are stained in blue (Hoechst), and immunostained proteins are shown in the following colors: green (ZO-1), magenta (β-Cat), yellow (E-cad), and red (Vim). Images were acquired using a Leica TCS SP8 confocal microscope. Scale bar: 30 μm. **(B)** Quantification of total fluorescence intensity for each marker from images in (A). Data are presented as mean ± SEM. Asterisk (*) indicates statistically significant differences (ANOVA, Tukey’s test, p < 0.05, n = 4).

To get further insight into cell junction integrity and organization, we performed immunofluorescence staining (IF) for ZO-1, β-catenin (β-cat), E-cadherin, and vimentin. The immunostaining revealed that in wt-CFTR expressing cells, ZO-1, β-cat, and E-cadherin were predominantly localized at the cell membrane in a continuous, linear pattern, indicative of well-organized epithelial junctions (Figure 2A). In contrast, staining for these markers in p.Phe508del-CFTR cells showed reduced membrane localisation and a disorganized and diffused cytoplasmic pattern, pointing to a disrupted junctional organisation and impaired polarization (Figure 2A). Moreover, quantification of ZO-1 fluorescence showed a significant increase in p.Phe508del-CFTR cells when compared to wt cells (Figure 2B), which is in line with the results obtained by WB. In p.Gly551Asp-CFTR cells, a similar, although less pronounced, diffuse staining pattern for ZO-1, β-cat, and E-cadherin was observed together with a trend towards increased ZO-1 fluorescence intensity, suggesting that only a partial compromise of junction integrity occurs with loss of CFTR ion transport, when it is present at the PM (Figures 2A,B). However, quantification of total fluorescence intensity revealed no significant differences for E-cadherin and β-cat across the three cell lines, consistent with WB data (Figure 2B). On the other hand, mesenchymal marker vimentin expression was detected in a higher number of cells in p.Phe508del-CFTR cells compared to both wt- and p.Gly551Asp-CFTR cells (Figures 2A,B). In conclusion, these IF results are consistent with the WB findings, underlining that the absence of membrane-localized CFTR exerts a stronger effect on junctional organization/integrity and mesenchymal marker expression than impaired CFTR ion transport alone.

### 3.2 Wound closure is delayed in CF cells

**FIGURE 3:**
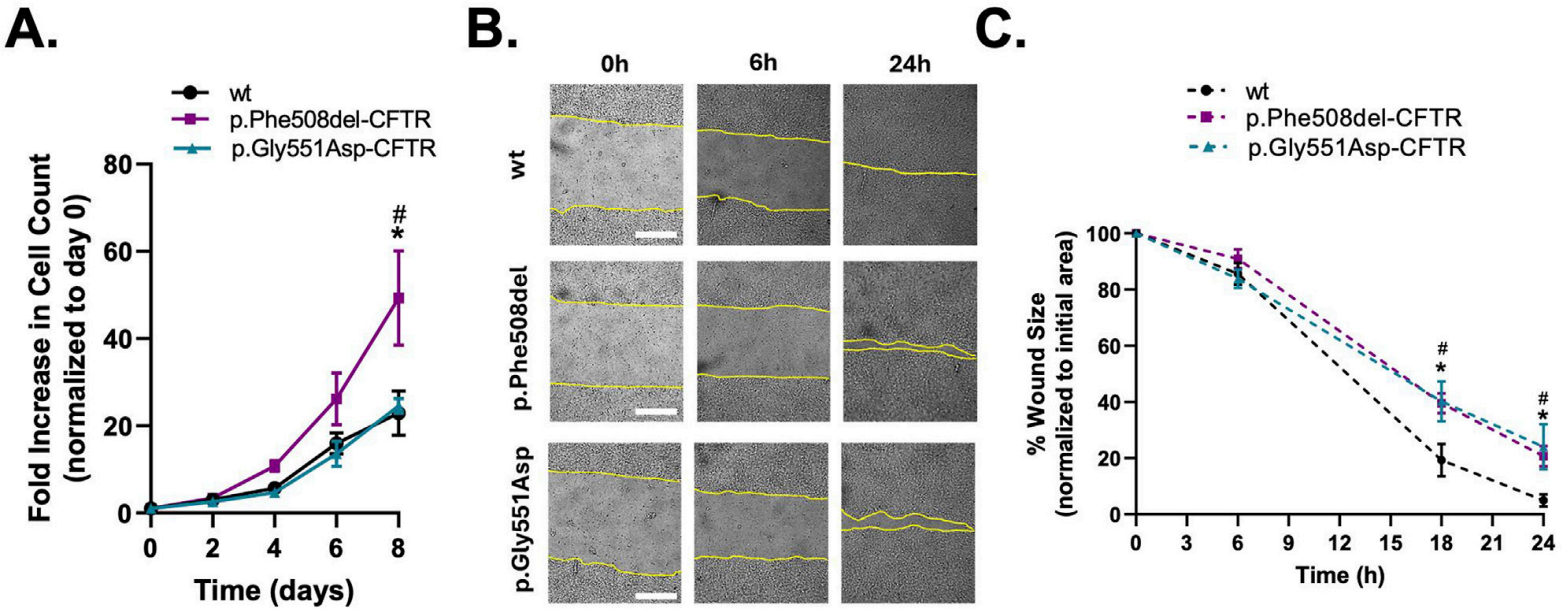
Proliferation and regeneration of wt-, p.Phe508del-, and p.Gly551Asp-CFTR CFBE cells. **(A)** Growth curve on non-polarized wt-, p.Phe508del-, and p.Gly551Asp-CFTR CFBE cells. Cells were harvested and counted every 2 days up to 8 days to assess the growth rate. The fold increase in cell number was calculated relative to the cell count at day 0. Data are presented as mean ± SEM. Asterisk (*) indicates significant difference (ANOVA, Tukey’s test, p < 0.05, n = 5). **(B)** Representative images of scratch wounds over 24 h on polarized CFBE cells. Live-cell imaging was performed. Wound segmentation is shown in yellow for clearer viewing. Scale bar represents 100 μm. **(C)** Wound closure analysis. Data is represented as a percentage of the initial area of the wound, mean ± SEM. Asterisk (*) and cardinal (#) indicates significant difference p.Phe508del and p.Gly551Asp vs. wt, respectively (ANOVA, Tukey’s test, p < 0.05, n = 5).

As previous studies have shown that CF cells display increased proliferation while wound closure (mostly migration) is impaired (Quaresma et al., 2020; Hajj et al., 2007; Trinh et al., 2012; Schiller et al., 2010), we sought to assess this here in the two CFBE cells expressing mutant CFTR in order to compare the impact of CFTR mislocalisation versus loss of function on these processes. Consistent with previous reports, CFBE cells expressing the p.Phe508del-CFTR variant exhibited a modestly higher proliferation rate during the initial days, which became statistically significant by day 8, when compared to both wt- and p.Gly551Asp-CFTR cells (Figure 3A). In contrast, proliferation rates of wt- and p.Gly551Asp-CFTR cells remained comparable, indicating that the mislocalisation and loss of CFTR at the PM, rather than loss of ion transport, drives an increased proliferation. In contrast, wound healing analysis also shows a delay in p.Gly551Asp-CFTR expressing cells, similarly to p.Phe508del cells, when compared to wt cells (Figures 3B,C). These data suggest that CFTR PM localisation is critical to control epithelial cell proliferation, whereas CFTR ion transport plays a more prominent role in regulating migratory responses.

### 3.3 CFTR modulators partially revert EMT phenotype in CF

**FIGURE 4:**
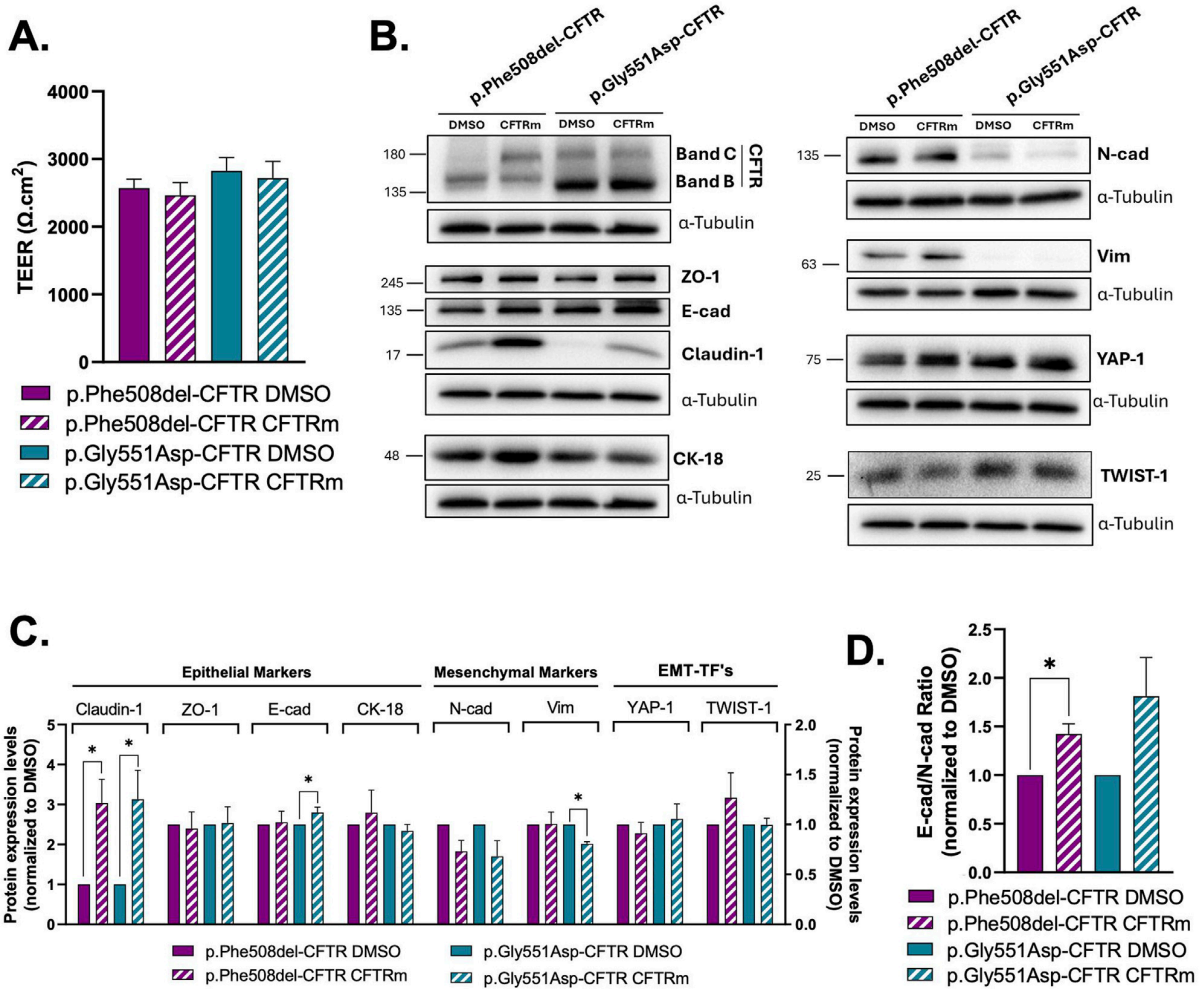
Effect of CFTR modulator (CFTRm) treatment on the epithelial/mesenchymal status of wt-, p.Phe508del-, and p.Gly551Asp-CFTR CFBE cells. **(A)** TEER measurements of polarized p.Phe508del- or p.Gly551Asp-CFTR CFBE cells treated with DMSO (as a vehicle control) or CFTRm. p.Phe508del-CFTR cells were treated with VX-445/VX-661/VX-770, and p.Gly551Asp-CFTR cells with VX-770 alone. Data are presented as mean ± SEM. Asterisk (*) indicates significant difference (unpaired Student’s t-test, p < 0.05, n = 8). **(B)** Representative WB images showing the expression of epithelial (CFTR, ZO-1, E-cad, CK-18 and claudin-1) and mesenchymal (N-cad and Vim) protein markers and EMT-TF’s (TWIST-1 and YAP-1) in polarized CFBE cells. α-tubulin was used as loading control. **(C)** Quantification by densitometry of the protein expression detected by WB in B. Left y-axis corresponds to claudin-1; right y-axis to all other markers. Data is first normalized to loading control, and then normalized to the DMSO condition, showed as mean ± SEM. Asterisk (*) indicates significant difference (Unpaired Student’s t-test, p < 0.05, n = 3). **(D)** E-cadherin and N-cadherin ratio, reflecting epithelial versus mesenchymal phenotype. Data are presented as mean ± SEM. Asterisk (*) indicates significant difference (Unpaired Student’s t-test, p < 0.05, n = 3).

Next, we aimed to determine how treatment with current CFTR modulators (CFTRm) reverses the EMT phenotype found in both cells expressing mutant CFTR. While p.Phe508del-CFTR cells were treated with the triple combination of two correctors (VX-445 and VX-661) and a potentiator (VX-770), p.Gly551Asp-CFTR cells were treated with VX-770 alone to restore ion transport. TEER measurements revealed no significant change in barrier function following treatment with CFTRm compared to DMSO-treated controls in both CF cells (Figure 4A). WB analysis revealed that claudin-1 expression was increased and thus rescued by CFTRm in both p.Phe508del- and p.Gly551Asp-CFTR cells (Figures 4B,C). Furthermore, in p.Phe508del-CFTR cells, treatment with the triple combination significantly increased the E-cadherin/N-cadherin ratio, while no changes were seen for ZO-1 expression (Figures 4B–D). Interestingly, VX-770 treatment in p.Gly551Asp-CFTR cells led to a slighty but significant increase in E-cadherin, a significant decrease in vimentin levels, and a tendency, albeit not significant, towards an increased E-cadherin/N-cadherin ratio (Figures 4B–D). However, TWIST-1 and YAP-1 expression levels were unaffected by treatment in both cell lines (Figures 4B,C). These results suggest that CFTRm treatment can partially rescue epithelial features but is not sufficient to fully restore the integrity of epithelial barrier in CFBE cells expressing either p.Phe508del- or p.Gly551Asp-CFTR.

### 3.4 TGF-β1 induces a more severe EMT phenotype in CF cells

**FIGURE 5:**
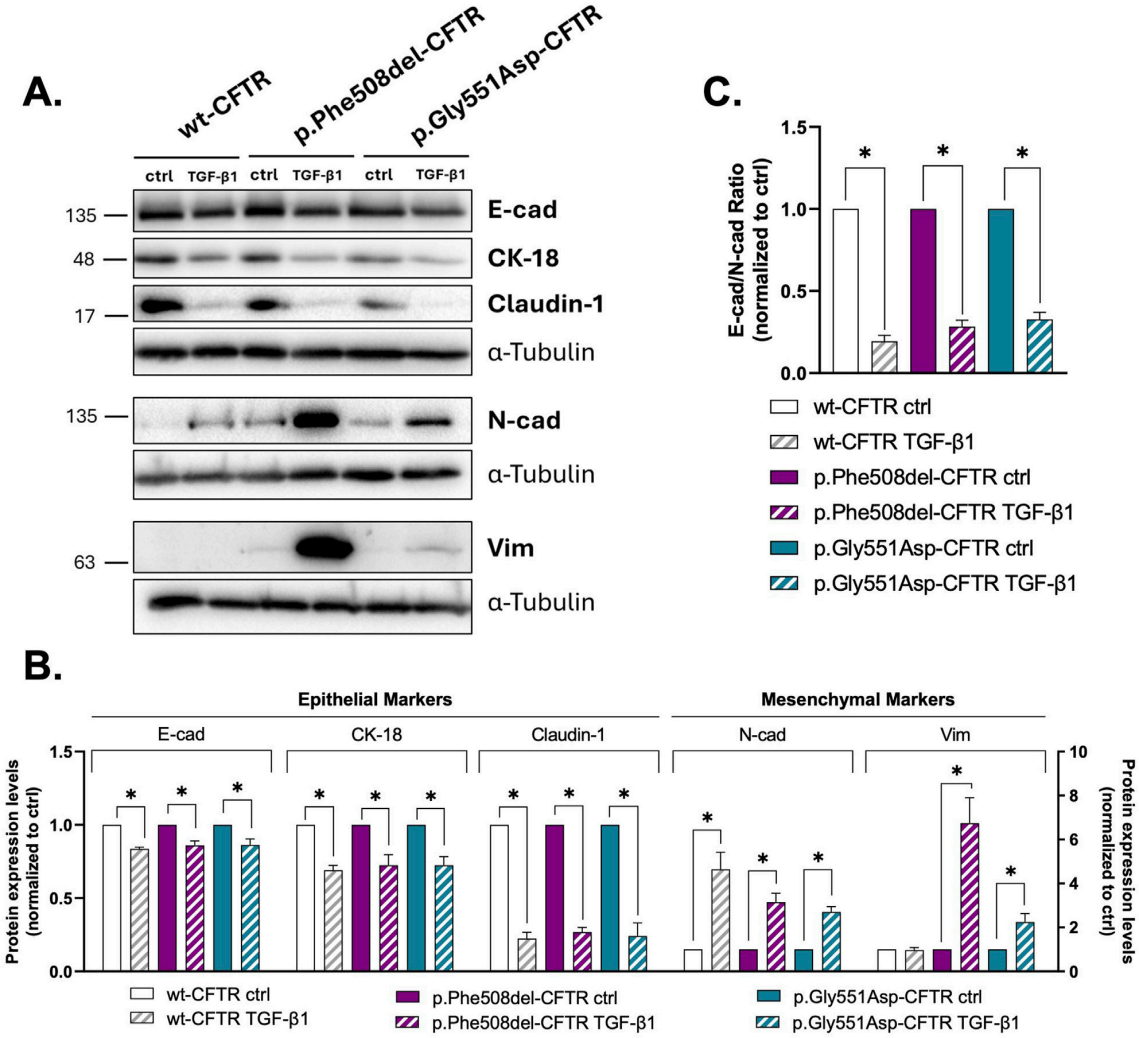
Effect of TGF-β1 treatment on the epithelial/mesenchymal status of wt-, p.Phe508del-, and p.Gly551Asp-CFTR CFBE cells. **(A)** Representative WB images showing the expression of epithelial (E-cad, CK-18 and claudin-1) and mesenchymal (N-cad and Vim) protein markers in polarized wt-, p.Phe508del- or p.Gly551Asp-CFTR CFBE cells treated with 10mM citric acid with 0.1% BSA (ctrl, as a vehicle control) or TGF-β1 (15 ng/mL). α-tubulin was used as loading control. **(B)** Quantification by densitometry of the protein expression detected by WB in A. Left y-axis corresponds to epithelial markers; right y-axis to mesenchymal markers. Data is first normalized to loading control, and then normalized to the ctrl condition, showed as mean ± SEM. Asterisk (*) indicates significant difference (Unpaired Student’s t-test, p < 0.05, n = 3). **(C)** E-cadherin and N-cadherin ratio, reflecting epithelial versus mesenchymal phenotype. Data are presented as mean ± SEM. Asterisk (*) indicates significant difference (Unpaired Student’s t-test, p < 0.05, n = 3).

Following our observation that loss of CFTR function alone results in a weaker EMT phenotype, we next evaluated how the two CFTR mutants affect EMT susceptibility/resilience. For this, polarized CFBE cells expressing wt-, p.Phe508del-, or p.Gly551Asp-CFTR were chronically treated with TGF-β1. TGF-β1 is a cytokine known to be elevated in CF tissues and furthermore is recognized as a major driver of EMT in airway epithelial cells (Xu et al., 2009; Harris et al., 2011). WB results revealed a significant decrease in key epithelial markers, namely, E-cadherin, CK18, and claudin-1, in both mutant and wt cell lines following TGF-β1 treatment, indicating a loss of epithelial characteristics regardless of the CFTR genotype (Figures 5A,B). Simultaneously, a robust increase in the mesenchymal marker N-cadherin could be observed in all CFBE cells, reflected by a significant reduction in the E-cadherin/N-cadherin ratio, which highlights the induction of EMT across all cell lines (Figure 5C). Interestingly, vimentin, another mesenchymal marker, was found to be upregulated only in cells expressing both CFTR mutants, whereas cells expressing wt-CFTR showed no significant change in vimentin levels after TGF-β1 exposure (Figures 5A,B). This finding suggests that CF cells, despite their different molecular defects, are more susceptible to TGF-β1-induced mesenchymal changes than wt-CFTR expressing cells.

### 3.5 CFTR inhibition alters junctional protein expression

**FIGURE 6:**
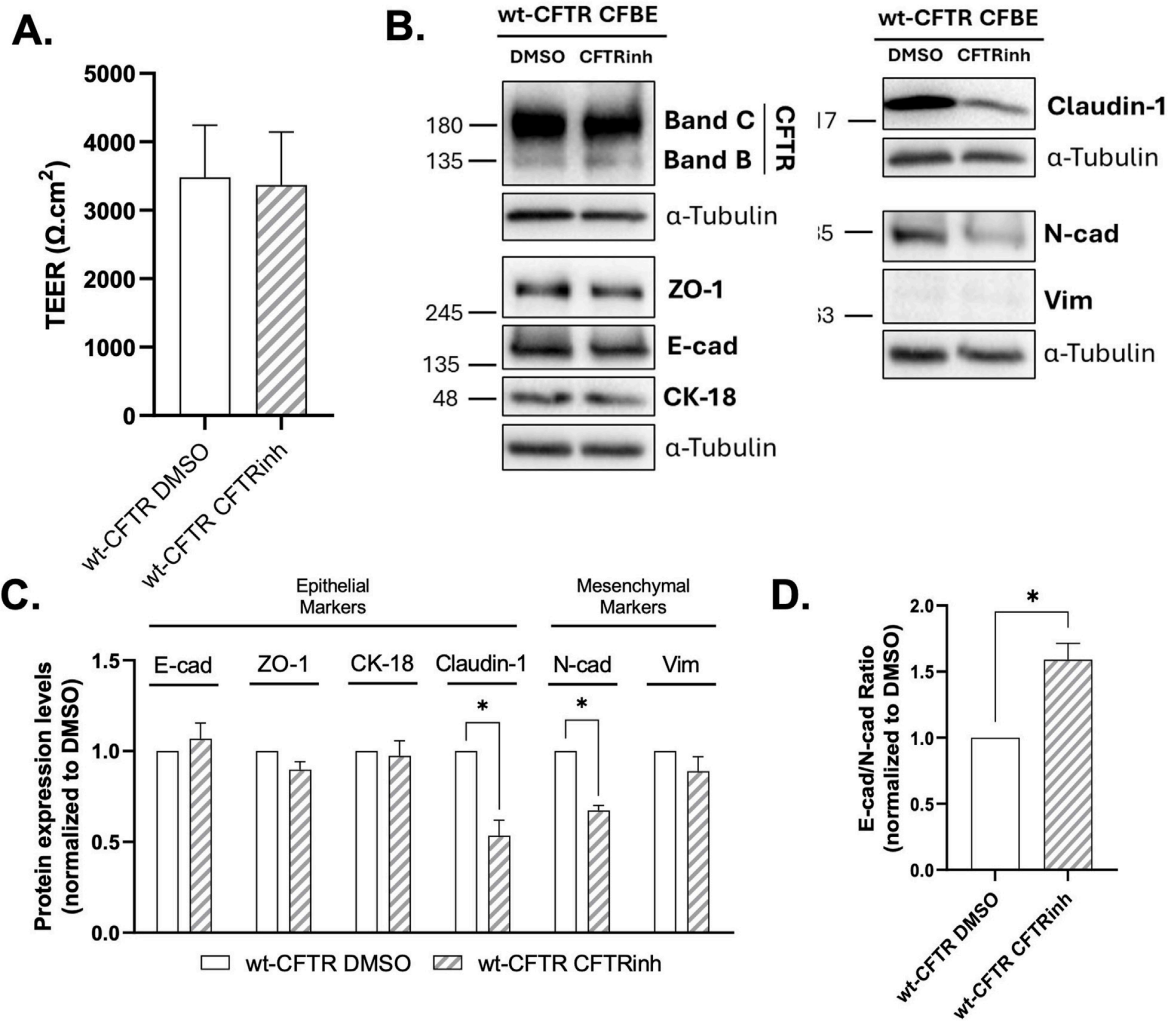
Effect of CFTRinh-172 treatment on the epithelial/mesenchymal status of wt-CFTR CFBE cells. **(A)** TEER measurements of polarized wt-CFTR CFBE cells treated with DMSO (as a vehicle control) or CFTRinh-172 (30 μM). **(B)** Representative WB images showing the expression of epithelial (CFTR, ZO-1, E-cad, CK-18 and claudin-1) and mesenchymal (N-cad and Vim) protein markers in polarized CFBE cells. α-tubulin was used as loading control. **(C)** Quantification by densitometry of the protein expression detected by WB in A. Data is first normalized to loading control, and then normalized to the DMSO condition, showed as mean ± SEM. Asterisk (*) indicates significant difference (Unpaired Student’s t-test, p < 0.05, n = 3). **(D)** E-cadherin and N-cadherin ratio, reflecting epithelial versus mesenchymal phenotype. Data are presented as mean ± SEM. Asterisk (*) indicates significant difference (Unpaired Student’s t-test, p < 0.05, n = 3).

To further assess the role of CFTR ion transport in maintaining epithelial integrity, we inhibited CFTR function in cells expressing wt-CFTR, thereby also functionally mimicking a gating-deficient state (as for p.Gly551Asp-CFTR) without affecting CFTR localisation. TEER measurements revealed no changes in cells treated with CFTRinh-172 (Figure 6A) suggesting that barrier function was still preserved. However, CFTRinh-172 led to a significant reduction in claudin-1 levels compared to DMSO treated cells (Figures 6B,C), which mirrors aspects of the p.Gly551Asp-CFTR phenotype (Figure 1B). However, in contrast to the latter, the E-cadherin/N-cadherin ratio significantly increased due to a decrease in N-cadherin levels, which indicates a relative shift toward an epithelial phenotype (Figure 6C). Notably, unlike the p.Gly551Asp-CFTR variant, CFTR inhibition did not lead to an increase in ZO-1 expression. These results show that acute loss of CFTR channel activity partially reproduces the junctional protein expression pattern seen in p.Gly551Asp-CFTR cells but does not fully recapitulate the EMT phenotype associated with this variant.

## 4 Discussion

The current study provides novel insight into the priming of the epithelium for partial EMT in CF by assessing the differential impact of CFTR variant-specific defects on epithelial barrier function, junctional protein organization, and susceptibility to EMT in airway epithelial cells.

We have previously shown that dysfunctional CFTR directly drives partial EMT in CF, independent of inflammation or infection, and identified EMTa-TFs TWIST1 and YAP1 as potential upstream regulators, both of which were found to be upregulated in CF cells (Quaresma et al., 2020; 2022). However, our previous work used mostly cells expressing p.Phe508del-CFTR, a variant that is retained in the ER thus failing to traffic to the PM. Our aim was to clarify whether the mere presence of CFTR at the PM without ion transport, would be sufficient to preserve epithelial integrity, or whether its ion transport function is also required. To investigate this, we used isogenic CFBE cell lines stably expressing either the ER-retained p.Phe508del variant (Class II), or the PM-localized but non-functional p.Gly551Asp variant (Class III) and wt-CFTR cells as controls (De Boeck and Amaral, 2016).

Our findings demonstrate that loss of CFTR ion transport is sufficient to prime EMT, albeit partially, but the absence of CFTR at the PM, results in a stronger mesenchymal phenotype and severe disruption of epithelial architecture and integrity. This was clearly observed when comparing p.Gly551Asp-CFTR cells with those expressing p.Phe508del-CFTR. The latter displayed markedly lower TEER, more disorganized junctional protein distribution, higher levels of mesenchymal markers, and lower E-cad/N-cad ratio, indicating that membrane-localized CFTR (p.Gly551Asp-CFTR) partially stabilizes epithelial architecture even in the absence of Cl^−^/HCO_3_^−^ transport.

While it is well established that functional CFTR supports tight junction assembly and barrier formation (Pankonien et al., 2022; LeSimple et al., 2010), our results indicate that CFTR trafficking to the PM itself also contributes to such epithelial organization, independent of its ion transport activity. This aligns with multiple studies showing that CFTR functions as a structural component of epithelial scaffolds (LeSimple et al., 2010; Ruan et al., 2014; Pankonien et al., 2022). In particular, CFTR interacts with PDZ-domain-containing scaffold proteins such as NHERF1 and ezrin that anchor it to the apical cytoskeleton and link it to the tight-junction protein ZO-1. These interactions are critical for organizing apical-basolateral polarity and for regulating cytoskeletal dynamics. When CFTR traffic is disrupted, as in the case of p.P508del, this network is destabilized, contributing to loss of epithelial polarity and barrier dysfunction. Moreover, a recent study by Rout-Pitt et al. (Rout-Pitt et al., 2025), which investigated EMT processes in CF rat models carrying either p.Phe508del- (class II) or a CFTR-KO (mimicking class I mutations), also suggests that the signalling pathways underlying EMT are CFTR mutation-dependent. Their results revealed that CFTR-KO cells, which completely lack CFTR, express higher levels of EMT-TF TWIST1 and mesenchymal marker N-cadherin but notably lower expression levels of type I collagen compared to p.Phe508del expressing cells, supporting our findings that the extent of the EMT state depends not only on CFTR function but also on its PM localisation.

Our proliferation and wound healing data show that loss of membrane-localized CFTR (p.Phe508del-CFTR) leads to abnormal proliferative signalling and impaired wound healing capacity. These observations are consistent with previous findings from our group and others (Quaresma et al., 2020; Trinh et al., 2012), which evidenced a role for CFTR in constraining proliferation and promoting wound repair. In contrast, the normal proliferation rate observed for p.Gly551Asp- but not for p.Phe508del-CFTR cells, is likely due to the mere physical presence of CFTR at the PM, albeit nonfunctional, which helps maintaining some epithelial structure and limits abnormal growth signals. These differential behaviours might also be partly explained by the induction of ER stress in p.Phe508del-CFTR expressing cells (Trouvé and Férec, 2025) as some studies suggest that chronic ER stress can drive abnormal proliferation, a phenomenon observed in cancer and stem cell systems (Zhang et al., 2024; Zheng et al., 2025). To conclude, the severity of the EMT phenotype previously reported by Quaresma et al. (2020) appears to depend on CFTR ion transport, but absence of PM localized CFTR further worsens the EMT state. Nevertheless, as we previously discussed (Quaresma et al., 2020), the EMT program is context-dependent and can present varying outcomes as cells may transition through various intermediate states between epithelial and mesenchymal phenotypes. While EMT is often linked to enhanced cell motility, particularly “full EMT” in cancer metastasis (Brabletz et al., 2018; Stuelten et al., 2018), the reduced wound healing found in our CF cells may instead reflect the partial EMT state we observe here, in which collective cell migration still occurs, but evidencing impairment due to disrupted tight junctions. Furthermore, although EMT is linked to reduced proliferation in certain contexts (Evdokimova et al., 2009; Lovisa et al., 2015), the increased proliferation found in p.Phe508del cells further indicates that the partial EMT state promotes proliferation while restricting migration. These findings are consistent with a context-specific partial EMT state in CF cells supporting the concept that EMT is not a binary process (Yang et al., 2020).

In concordance with our previous study (Quaresma et al., 2020), the present results reveal that CFTRm can partially reverse EMT features in CF epithelial cells, but they fail to fully restore epithelial integrity. In both p.Phe508del- and p.Gly551Asp-CFTR models, CFTRm treatment increased the expression of tight-junction protein claudin-1, which is indicative of some level of restoration of airway epithelial integrity (Günzel and Yu, 2013; Kojima et al., 2013). Upon CFTRm, treatment mesenchymal markers in p.Phe508del-CFTR cells remained elevated, however, a modest shift towards an epithelial phenotype was observed, as seen by an increased E-cad/N-cad ratio (Loh et al., 2019). However, TEER values remained unchanged in both CF cell lines, and high levels of key EMT regulators TWIST1 and YAP1 were unaffected, indicating that the current modulator therapies are insufficient to fully reverse EMT phenotype driven by CFTR dysfunction. This also suggests that additional therapeutic strategies targeting EMT pathways may be necessary for full epithelial repair in CF.

To further dissect the role of CFTR in regulating EMT susceptibility, we assessed the epithelial sensitivity/resilience to chronic TGF-β1 stimulation. Consistent with elevated TGF-β1 levels reported in CF airways (Xu et al., 2009; Harris et al., 2011), chronic exposure to this cytokine triggered a robust EMT response across all CFBE cell lines, characterized by a decrease of epithelial markers (E-cadherin, CK18, claudin-1) and increased N-cadherin expression. However, the extent of EMT progression varied between cells and revealed a variant-specific susceptibility to TGF-β1-induced EMT, with wild-type CFTR-expressing cells showing greater resistance to mesenchymal reprogramming compared to both CF cells. Vimentin expression, for example, remained unchanged in wt-CFTR cells but was significantly upregulated in both p.Phe508del- and p.Gly551Asp-CFTR cells compared to no treatment. These findings indicate that functional CFTR confers protection against inflammatory and pro-fibrotic EMT, likely by maintaining structural and signalling networks at the apical PM. This is supported by a study showing that wt-CFTR suppresses TNF-α–induced NF-κB–mediated inflammation, although it does not directly assess epithelial integrity (Hunter et al., 2010). Importantly, among the two CFTR variants studies here, cells expressing p.Phe508del-CFTR showed a significantly higher increase in vimentin expression upon TGF-β1 treatment compared to p.Gly551Asp-CFTR cells, supporting the fact that the presence of CFTR at the PM provides partial resistance to EMT progression, even in the absence of ion transport. Consistent with this, clinical data from individuals homozygous for p.Gly551Asp show reduced lung damage than individuals carrying p.Phe508del/p.Phe508del, further suggesting that CFTR localisation alone contributes to the preservation of epithelial integrity in the airways (Comer et al., 2009).

To confirm that the loss of CFTR ion transport alone contributes to junctional protein dysregulation, we inhibited CFTR function in wt-CFTR expressing cells, somewhat mimicking p.Gly551Asp. While this treatment partially recapitulated aspects of the p.Gly551Asp-associated phenotype such as reduced claudin-1 levels, it also unexpectedly reduced the mesenchymal marker N-cadherin. Moreover, it did not alter ZO-1 expression or TEER levels. Although the effects of acute CFTR inhibition do not fully replicate the phenotype observed in p.Gly551Asp-CFTR expressing cells, the observed downregulation of claudin-1 highlights that disruption of Cl^−^ and HCO_3_^−^ transport mediated by CFTR impacts tight junction integrity, an early hallmark of EMT (Lamouille et al., 2014). Importantly, the 72-h CFTR inhibition rather represents an acute and reversible perturbation and therefore cannot fully model the chronic, constitutive ion transport defect present in p.Gly551Asp-CFTR cells. Nonetheless, these data support our results that CFTR at the PM contributes to maintaining epithelial integrity to some extent, while also indicating that ion transport function itself provides an additional protective role.

## 5 Conclusion

This study demonstrates that CFTR variant-specific defects distinctly influence epithelial barrier integrity, junctional protein expression, and EMT susceptibility in airway epithelial cells. Our results show that the mere physical presence of CFTR at the PM, even without mediating ion transport, already contributes to the maintenance of some epithelial organization, while also providing partial protection against EMT progression, e.g., caused by TGF-β1. In contrast, the absence of CFTR from the PM, as seen in p.Phe508del-CFTR, leads to a stronger EMT phenotype. While CFTRm partially restored tight junction components, they failed to fully rescue barrier integrity and to completely reverse the mesenchymal phenotype, particularly in CF cells expressing p.Phe508del-CFTR. The enhanced sensitivity of CFTR mutant cells to TGF-β1-induced EMT further implicates CFTR dysfunction in airway remodelling and fibrosis progression. Our findings suggest that, in addition to current modulator therapies, additional therapeutic strategies aimed at preventing EMT progression are likely necessary to improve long-term outcomes in CF, particularly given the increased cancer risk reported for people with CF.

## Data Availability

The original contributions presented in the study are included in the article/Supplementary Material, further inquiries can be directed to the corresponding authors.

## Supplementary material

SUPPLEMENTARY FIGURE S1 Functional analysis of wt-/p.Phe508del-/ p.Gly551Asp-CFTR CFBE cells by Ussing Chamber. (Left) Representative original short-circuit current (Isc) recordings from Ussing Chamber experiments, measuring CFTR-mediated chloride secretion as transepithelial voltage (Vte). Forskolin (Fsk) was applied apically to activate CFTR, followed by Genistein. Recordings are shown for untreated wt- and p.Gly551Asp-CFTR CFBE cells (panels **A** and **B**), and for p.Phe508del-CFTR CFBE cells pre-treated with either DMSO or 5 μM VX-661 in combination with 3 μM VX-445 (panel **C**). CFTRinh-172 was added at the end to confirm CFTR-specific activity. (Right) Quantification of equivalent short-circuit currents (Isc-eq) corresponding to responses to Fsk, Genistein+Fsk, VX-445/661/Genistein+Fsk, and CFTRinh-172. Data are presented as mean ± SEM (n = 3).
