# Supplementary figures and images for: CFTR ion transport deficiency primes the epithelium for partial epithelial-mesenchymal transition in cystic fibrosis
Source: Front Pharmacol. 2025 Aug 20;16:1655479. doi: 10.3389/fphar.2025.1655479 (PMC12405223; doi:10.3389/fphar.2025.1655479)

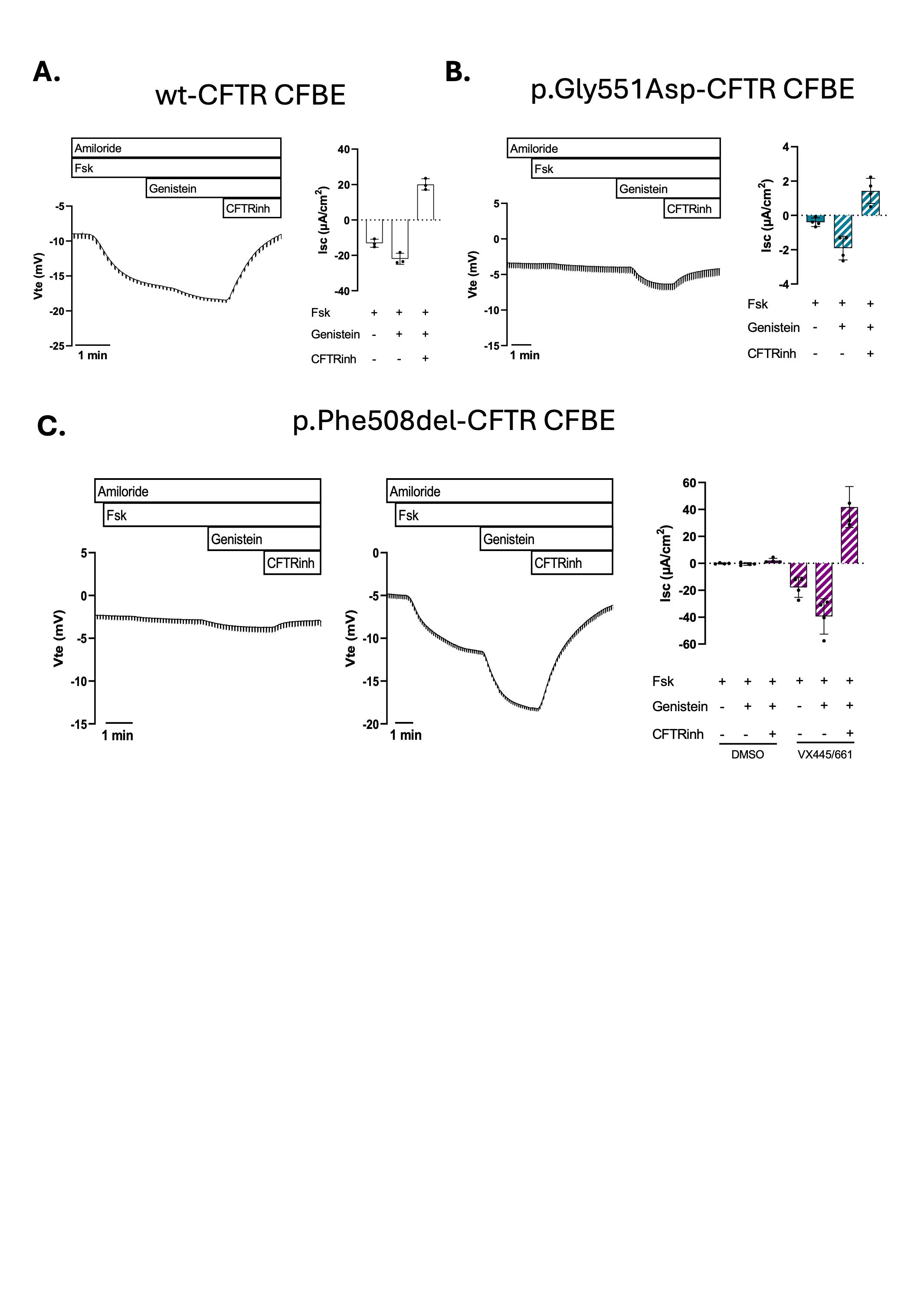

Supplement: Supplementary file 1 [file Image1.jpeg]
